# Modulation of Human Muscle Spindle Discharge by Arterial Pulsations - Functional Effects and Consequences

**DOI:** 10.1371/journal.pone.0035091

**Published:** 2012-04-17

**Authors:** Ingvars Birznieks, Tjeerd W. Boonstra, Vaughan G. Macefield

**Affiliations:** 1 School of Science and Health, University of Western Sydney, Sydney, New South Wales, Australia; 2 Neuroscience Research Australia, Sydney, New South Wales, Australia; 3 Black Dog Institute, Sydney, New South Wales, Australia; 4 School of Medicine, University of Western Sydney, Sydney, New South Wales, Australia; 5 School of Medical Sciences, University of New South Wales, Sydney, New South Wales, Australia; 6 School of Psychiatry, University of New South Wales, Sydney, New South Wales, Australia; Georgia State University, United States of America

## Abstract

Arterial pulsations are known to modulate muscle spindle firing; however, the physiological significance of such synchronised modulation has not been investigated. Unitary recordings were made from 75 human muscle spindle afferents innervating the pretibial muscles. The modulation of muscle spindle discharge by arterial pulsations was evaluated by R-wave triggered averaging and power spectral analysis. We describe various effects arterial pulsations may have on muscle spindle afferent discharge. Afferents could be “driven” by arterial pulsations, e.g., showing no other spontaneous activity than spikes generated with cardiac rhythmicity. Among afferents showing ongoing discharge that was not primarily related to cardiac rhythmicity we illustrate several mechanisms by which individual spikes may become phase-locked. However, in the majority of afferents the discharge rate was modulated by the pulse wave without spikes being phase locked. Then we assessed whether these influences changed in two physiological conditions in which a sustained increase in muscle sympathetic nerve activity was observed without activation of fusimotor neurones: a maximal inspiratory breath-hold, which causes a fall in systolic pressure, and acute muscle pain, which causes an increase in systolic pressure. The majority of primary muscle spindle afferents displayed pulse-wave modulation, but neither apnoea nor pain had any significant effect on the strength of this modulation, suggesting that the physiological noise injected by the arterial pulsations is robust and relatively insensitive to fluctuations in blood pressure. Within the afferent population there was a similar number of muscle spindles that were inhibited and that were excited by the arterial pulse wave, indicating that after signal integration at the population level, arterial pulsations of opposite polarity would cancel each other out. We speculate that with close-to-threshold stimuli the arterial pulsations may serve as an endogenous noise source that may synchronise the sporadic discharge within the afferent population and thus facilitate the detection of weak stimuli.

## Introduction

When the left ventricle of the heart ejects blood into the aorta the resultant pulse wave travels rapidly through the arterial system and reaches tissues throughout the body. Thus, mechanoreceptors inevitably become subjected to arterial pulsations when located in highly vascularised tissues. For example, almost half of all tactile afferents innervating the fingertips show cardiac modulation in some form and for some afferents even respiratory rhythmicity could be discerned [1], [2]. Similarly, arterial pulsations are known to modulate the discharge activity in muscle spindles [3]–[7], and sometimes are capable of driving muscle spindle discharge, spindle firing being time locked to the arterial pulse in the absence of ongoing background activity.

While McKeon and Burke [4] first described this phenomenon in recordings of human muscle spindles in a sample of 25 afferents, they only found three endings that were driven by the arterial pulse – the majority showed cardiac modulation that, the authors conclude, “are unlikely to be eliminated in the summed activity forming the population response.” One of the main objectives of these earlier studies was to investigate whether a synchronised response to arterial pulsations would compromise the capacity of spike-triggered averaging to measure the strength of synaptic connections between muscle spindle afferents and the spinal motoneurones [3], [8], [9]. Less attention has been drawn to the physiological consequences of such synchronised modulation by arterial pulsations. McKeon and Burke [4] suggested that that the arterial pulse could be a significant contributor to the discharge variability of muscle spindles and should be present in the population response, thereby limiting the information capacity of muscle spindle afferents. However, we do not know whether changes in either the magnitude of arterial pulsations, or the sensitivity of muscle spindles, during various physiological conditions translates into a physiologically significant change in discharge variability that may influence proprioceptive function. A reduction in the proprioceptive accuracy may require an increase in the co-activation of agonist and antagonist muscles to guide the limb to the intended action's endpoint. Indeed, it is perhaps not surprising that changes in proprioceptive accuracy have been associated with musculoskeletal disorders and pain [10]. If hemodynamic effects influence muscle spindle discharge it is important to know the magnitude of this effect, as it might be one of the pathways that link pain, emotional stress, exercise and fatigue with proprioceptive function and thus sensorimotor control. Therefore, one of the central objectives of this study was to examine whether various physiological stressors have the capacity to influence the amount of physiological noise induced in muscle spindles by arterial pulsations and, thereby, potentially lead to clinically significant adverse consequences.

The variability of muscle spindle discharge is often ascribed to fusimotor activity; however, it generally does not accurately correlate with the level of fusimotor drive [5]. Changes in muscle hemodynamic parameters may be another important constituent of the variability in muscle spindle discharge, especially in animals with high heart rate. The functional consequences of physiological noise have recently gained increasing attention, as intermediate levels of noise may actually facilitate sensory processing [11]. Hence, a systematic assessment of the contribution of arterial pulsations to discharge variability is required to understand its role in the sensory function of muscle spindles.

In the current study we characterize the modulatory effects of arterial pulsations on the spontaneous discharge of human muscle spindles, both at rest and during physiological challenges and manoeuvres. Discharge modulation was quantified in the time and frequency domains to allow detailed comparisons of the effects of arterial pulsations across physiological conditions. In the first experiment, we examined changes in discharge modulation caused by an inspiratory-capacity apnoea, a manoeuvre known to have a profound effect on hemodynamic parameters and muscle sympathetic nerve activity without apparent activation of fusimotor neurones in humans [12]. While there is no evidence of a direct modulatory effect of sympathetic nerve activity on muscle spindle discharge in humans [12], such effects have been demonstrated in animals [13], [14] and might need to be evaluated in the current study. In a second experiment we investigated whether muscle spindle modulation is changed during acute pain, induced by intramuscular or subcutaneous injection of hypertonic saline. Any change in spindle activity during pain could be caused by local changes in perfusion or by nociceptive reflexes involving fusimotor neurones. It has been previously demonstrated in animal experiments that pain can increase spindle sensitivity [15], but this could not be replicated in humans [16]. In the third experiment we investigated whether cardiac modulation affects spindle discharge during muscle contraction, which is known to increase the discharge of fusimotor neurones. Finally, we will summarise the potential functional consequences such modulation may have on the physiological function of muscle spindles. The robustness of the effect we observed indicates that fluctuations in blood pressure during various physiological conditions have a limited capacity to alter signalling capacity of the afferent population through this mechanism. We also suggest that intermediate levels of physiological noise in some situations could probably facilitate the detection of weak stimuli.

## Materials and Methods

### Ethics approval

Data were obtained from 40 healthy subjects, 24 males and 16 females ranging in age from 18 to 42 years. Each subject provided informed written consent to the procedures, which were approved by the human research ethics committee of the University of New South Wales and conformed to the Declaration of Helsinki.

### Microneurography

The common peroneal nerve was located at the fibular head by palpation and electrical stimulation via a surface probe. A tungsten microelectrode (Frederick Haer & Co. Inc., Brunswick, ME, USA) was inserted percutaneously into a motor fascicle of the nerve. Fine manipulation of the microelectrode to isolate single afferents was then performed using auditory feedback of the neural activity while providing mechanical stimuli to the pretibial muscles and tendons of the leg. Unitary recordings were made from muscle spindle afferents. Neural activity was amplified (gain 1×10^4^), and filtered at 0.3–3.0 kHz, 50 Hz notch (ISO-80, World Precision Instruments, USA) and digitised at 10–20 kHz sampling rate, depending on bandwidth required by other channels to be recorded. All electrophysiological data were recorded on a computer-based data acquisition system (PowerLab 16SP, ADInstruments, Bella Vista, Australia).

Recorded nerve activity was exported and saved in Igor Pro 5 format (Wavemetrics, USA). Single spike recognition and discharging activity parameter analyses were performed using custom software developed using Igor Pro 5. Discharge activity of single muscle spindle afferents were visually scrutinised during the whole time course of the experiment to detect any inconsistencies and artefacts. Instantaneous discharge rate was calculated as inverse of inter-spike intervals between two successive nerve impulses. R-wave triggered averaging of muscle spindle instantaneous discharge rate traces was performed on blocks of data with a minimal duration of 30 s. The peak of the R-wave was used as a trigger point.

### Identification and sample of muscle spindle afferents

Identification of the muscle spindle afferents was based on the responses to stretch of the muscle along its line of action, palpation and vibration over the muscle tendons and belly and weak voluntary contractions. Single muscle afferents were identified as group Ia or II spindle afferents according to criteria previously described [17]. Briefly, primary afferents show high dynamic stretch sensitivity, demonstrate an irregular spontaneous or volitionally maintained discharge, and exhibit an off-response at the point of abrupt relaxation following a slow ramping isometric contraction. In contrast, secondary afferents usually exhibit a more regular tonic discharge [18], decelerate during an unloading contraction and do not exhibit an off-response at the termination of a voluntary ramp-and-hold contraction.

### Measurement of hemodynamic parameters

Continuous blood pressure was measured non-invasively using radial arterial tonometry (Colin NIBP, Colin Corp, Japan) and heart rate (HR) via standard Ag–AgCl electrocardiogram (ECG) chest electrodes. Respiration was recorded with a strain gauge transducer attached to a strap around the chest (Pneumotrace, UFI, Morro Bay, CA, USA). The ECG signal was used to generate R-wave triggered averages.

### Electromyography

Surface EMG was recorded via Ag/AgCl electrodes over the tibialis anterior (TA), extensor digitorum longus (EDL) and peronei muscles to confirm that the muscles were relaxed. The surface electrodes were placed over the muscle belly with an inter-electrode distance of 20–25 cm and in optimal positions for picking up surface recordings from the majority of the muscles active fibres. To ensure that muscles remained relaxed in the first two experiments in which spontaneously active afferents were investigated, the RMS filtered EMG signal was inspected visually and total surface area under the EMG curve was calculated and compared with baseline.

### Physiological conditions

#### Apnoea

Subjects were asked to perform an inspiratory-capacity apnoea, in which subjects hold their breath at maximal lung volume against a closed glottis for 30–40 s [19].

#### Nociceptive stimulation

Two 23 G cannulae were inserted; one ∼1 cm into the belly of the tibialis anterior muscle of the same leg form which the spindle afferent was being recorded; the other subdermally into the overlying skin ∼5 mm away. Intramuscular bolus injection of 5% hypertonic saline (0.5 ml) caused sensations of deep, dull and diffuse pain, whereas subdermal injections of 5% hypertonic saline (0.2 ml) caused sensations of localised, sharp burning pain. After an appropriate baseline period was recorded, injections were made at unexpected times and in quasi-random order. Subjective pain level was evaluated on a visual-analog scale from 0 to 10, where 0 was described as “no pain” and 10 as “the worst pain ever experienced by the subject”. Subjects indicated instantaneous pain level by a labelled potentiometer dial.

#### Voluntary contraction

Subjects were asked to dorsiflex their ankle and gently apply force against force transducer and keep it at a constant level set to activate the muscle spindle at the rate of about 10 imp/s. Muscle force production was displayed on a computer monitor during voluntary muscle contractions for visual feedback to the subject.

### Analyses of pulse-wave effects on muscle spindle discharge rate

The pulse wave effect was estimated both in the time and frequency domain [20]. In the time domain the effect was determined by R-wave triggered averaging. Instantaneous discharge rate traces were cut into segments between each of two consecutive R-wave peaks (RR intervals). Segments containing discharge rates that were more than three standard deviations (SD) different from the mean were removed to exclude artefacts and errors in automatic spike recognition. The remaining data were Fisher z-transformed to have zero mean and unit variance. If *n* is the number of data points in all artefact-free segments, this is achieved with 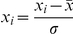, where 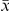 is the mean and 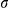 the standard deviation over all data points 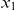, …, 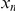. The normalized data segments were then averaged with respect to the R-wave triggers. Note that due to normalization, the variance of the average segment must lie between 0 and 1. If the modulation of discharge rate is uncorrelated across segments, then the variance will be 0. If all segments are identical (fully correlated), then the variance of the average segment will be exactly 1. Otherwise, the variance will lie between 0 and 1. Hence, the variance of the R-triggered average quantifies the amount of the total variance explained by the effect of arterial pulsations (pulse wave modulation).

To statistically test whether this modulation is caused by the pulse wave or by random events not related to cardiac rhythmicity, surrogate data were generated by circular permutation, i.e. each data segment was shifted a random number of samples before re-computing the average. A set of 1000 surrogates was generated. Because only the alignment of segments was changed, the variance of the surrogate averages represents the explained variance under the null hypothesis. A straightforward statistical comparison between this null distribution and the value derived from the experimental data then permits formal testing of the null hypothesis. This nonparametric technique of generating a null distribution has a well-established role in statistics cf [21], [22]. The pulse wave modulation was considered statistically significant if the explained variance was larger than 95% (p<0.05) of the surrogate distribution.

In the frequency domain the pulse wave effect was assessed by means of the power spectral density of the discharge rate traces. The power spectral density is the Fourier transform of the autocorrelation function and can be used to test for modulations at the heart rate frequency. Power spectral density was estimated using Welch's periodogram method using overlapping Hanning windows (window length, 7 s; overlap 3.5 s) [23]. To test for significant periodicity in the discharge rate, a set of 1000 surrogates were generated. To this end, the order of the spikes was locally permuted while leaving the inter-spike intervals the same, i.e. destroying possible temporal dependencies while leaving the mean and variability of discharge rate unchanged [24]. For each surrogate signal the power spectrum was computed and this surrogate distribution was then used to define the 95% confidence interval.

### Principal component analysis

Principal component analysis (PCA) was employed to compare R-wave averages and power spectra across muscle spindles and to test for pulse wave modulation at the population level. PCA has commonly been used for unbiased statistical comparison of multivariate data across conditions [25]–[27]. Separate PCAs were conducted for R-wave averages and power spectra. The time and frequency axes were renormalized to compare discharge modulation across recordings with varying heart rates. The time axes of the averages were resampled using linear interpolation such that each RR interval contained an equal number of samples. The frequency axes of the power spectra were transformed from a linear scale to one relative to the heart rate frequency spanning 0 to 6 times the heart rate frequency.

The broken-stick method was used to determine the number of principal components that were considered relevant for interpretation [28]. Of those principal components, the data were projected onto the eigenvector to determine the dominant features in the R-wave averages and power spectra. To determine the stability of those projections, the standard errors of each data point in the projection were estimated through 1000 bootstrap samples [29]. Bootstrap samples were generated using sampling-with-replacement and PCA was recalculated for each bootstrap sample. The ratio of the original projection to the bootstrap standard error is approximately equivalent to a z-score if the bootstrap distribution is normal [29]. Data points in the projection were considered stable if their z-scores were larger than 2.58 (p<0.01). Finally, eigenvector coefficients of significant principal components were compared across pain conditions and regressed against other variables to investigate correlations with the amount of discharge modulation [26],[27].

### Statistical analysis

A combination of parametric and nonparametric statistics was used to describe the data and test for statistical significance [30]. To describe the prevalent effect characteristic for single muscle spindle afferents during normal conditions we primarily report median values. By contrast, we report mean values in the context of possible physiological effects because, regardless of skewed distributions, means better reflect functional consequences of converging inputs from a population of muscle spindles. The Wilcoxon matched-paired signed-ranks test was used to detect the prevailing direction of differences within population. In this test each afferent was represented by pair of values representing two different conditions – control versus apnoea or pain. The Pearson coefficient of correlation (r) was used as measure of correlation. As a nonparametric alternative and measure of correlation, we used the Spearman rank correlation coefficient (r_s_). In all tests, the level of probability selected as significant was p<0.05. Unless otherwise indicated, population estimates are presented as mean values.

## Results

### Afferent sample

Unitary recordings were made from a total of 75 muscle spindle afferents in 40 subjects. Discharge activity in 58 muscle spindle afferents showing spontaneous activity was recorded from relaxed leg muscles while voluntary muscle contraction was required to activate the remaining 17 afferents. Based on behavioural criteria 50 afferents were classified as supplying primary endings (group Ia afferents) and 25 as supplying secondary endings (group II afferents).

### Muscle spindle firing driven by arterial pulsations

**Figure 1:**
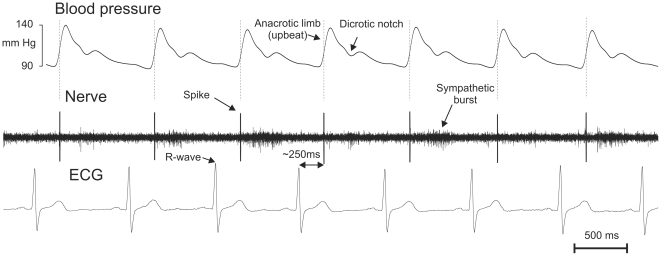
Example of muscle spindle discharge locked to the arterial pulsations. This afferent responded with one single spike at the early part of the upbeat of pulse wave and about ∼250 ms following R-wave in ECG signal. Note also muscle sympathetic burst activity in nerve signal.

**Figure 2:**
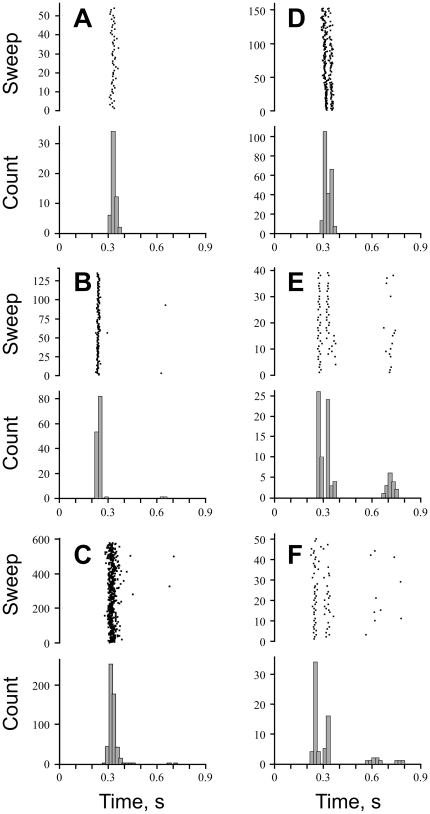
Pulse-wave driven muscle spindles. Each panel shows post stimulus time histogram at the bottom and raster sweeps of single cardiac cycles on top. **A–C**, Afferents typically responded with one single spike phase-locked to the cardiac cycle. **D–F**, Afferents responding with two spikes at the time corresponding to the pulse wave upbeat. A third spike corresponding to the time of dicrotic notch is generated by afferents depicted in **E** and **F** (see also **B**&**D**). Each bar indicates total count of spikes falling within corresponding 0.02 s time bin. Each dot in raster plot indicates one spike.

In total, we encountered seven muscle spindles discharging at a relatively low discharge rate (≤3 imp/s), apparently driven by arterial pulsations, e.g., showing no other spontaneous activity than spikes generated with a clear cardiac rhythmicity. Four muscle spindles fired one spike per cardiac cycle, corresponding approximately to the ascending section of the pulse wave (anacrotic limb or upbeat), which occurs about 250 ms after the peak of the R-wave of the ECG (Fig. 1). R-wave triggered post-stimulus time histograms revealed that every spike generated in these afferents was locked to the R-wave and thus exclusively driven by the arterial pulse, as shown for the three units in Fig. 2A–C. We also encountered 3 muscle spindles that fired two or three spikes per cardiac circle. As shown in Figure 2D–F, the second spike usually closely followed the first. Two of those muscle spindles (Fig. 2E–F) showed a second cluster centred at the latency corresponding to the dicrotic notch of the pulse wave (cf. Fig. 1).

### Phase locking of ongoing spindle discharge

**Figure 3:**
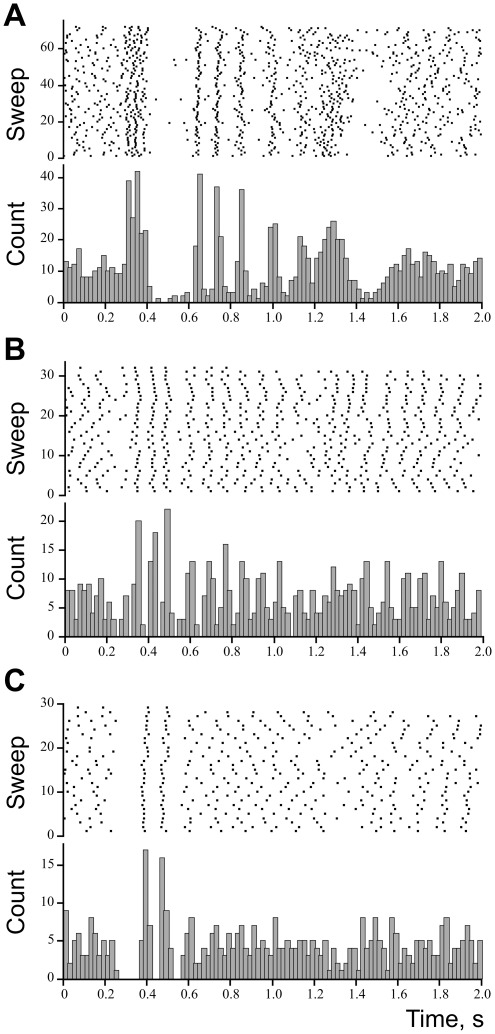
Phase-locking and resetting of ongoing muscle spindle discharge. For further details see text and legend of Fig. 2.

For the majority of spontaneously active afferents the ongoing discharge was primarily evoked by muscle stretch properties other than the pulse wave. However, the effects of cardiac rhythmicity could be discerned also in those afferents. The most pronounced effect was phase locking of individual spikes. In the literature such kind of behaviour is referred to as “*resetting*,” and is described as an afferent that “discharged or failed to discharge at a fixed interval after the pulse” [4]. Thus, such behaviour was predicted and searched for [4], but has not been previously found. No formal criteria have been developed to distinguish such afferents, therefore we relied on visual inspection of spike alignment in rasterplots and peak-and-trough patterns in density histograms. Such behavior is rarily seen in muscle spindle afferents, however three afferents clearly stood out. For example, the primary spindle afferent shown in Fig. 3A discharged spontaneously at a low rate, around 10 imp/s. At 300 ms after the R-wave, corresponding to the ascending portion of the pulse wave, its instantaneous discharge rate exceeded 30 imp/s. After the peak there was a silent period of about 200 ms when the discharge virtually ceased. This is a very typical characteristic of dynamically sensitive group Ia muscle spindle afferents – they usually exhibit a silent period following an excitatory stimulus [31], [32]. Spiking activity resumed after 650 ms; this latency was constant across trials. In this subject, the R-R interval was 0.95 s and, due to the heart rate variability, the raster plot showed substantial jitter at the onset of the next systolic wave. Thus, in this case, phase locking of spiking activity resulted from post-stimulus depression.

In another Ia afferent phase locking was achieved through a different mechanism. As shown in the example illustrated in Fig. 3B, phase locking occurred because of the excitatory effect of the upstroke of the pulse wave (Fig. 3B). Three spikes generated at the time of the upstroke of the pulse wave showed slightly increased discharge rate and tight phase locking regardless of the timing of the preceding spike. The first inter-spike interval following the triplet was slightly prolonged, after which regular spiking activity resumed. R-triggered raster plots showed that these spikes were well aligned, although there was more jitter than during the pulse wave period.

By contrast, the third phase locking mechanism in spontaneously active afferents was based on *inhibition* by the upstroke of the pulse wave (Fig. 3C). After being silenced for about 150 ms, approximately 250 ms after the R-wave the afferent resumed its firing with two phase-locked spikes. The following spikes showed considerable jitter and phase locking was not maintained.

### Muscle spindle discharge modulated by arterial pulsations

In previous sections we demonstrated recordings from muscle spindles whose spiking activity was essentially driven by arterial pulsations and spontaneously active afferents in which some spikes apparently became phase-locked to the arterial pulsations. However, for the majority of tonically active muscle spindles – activated by the existing static stretch of the relaxed muscle – a more subtle modulation of discharge activity by the pulse wave was present. In these afferents, a modulation pattern could be discerned in the R-wave triggered average firing rates. This reflected a tendency to increase or decrease their instantaneous discharge rate while individual spikes were not locked to the arterial pulsations. The following analyses were undertaken to identify a proportion of afferents with significant pulse wave modulation, the proportion of total variance explained by the pulse wave, and the common modulatory pattern in the firing of muscle spindle afferents (see Methods).

#### Cardiac rhythmicity detected in the background discharge of muscle spindle afferents

**Figure 4:**
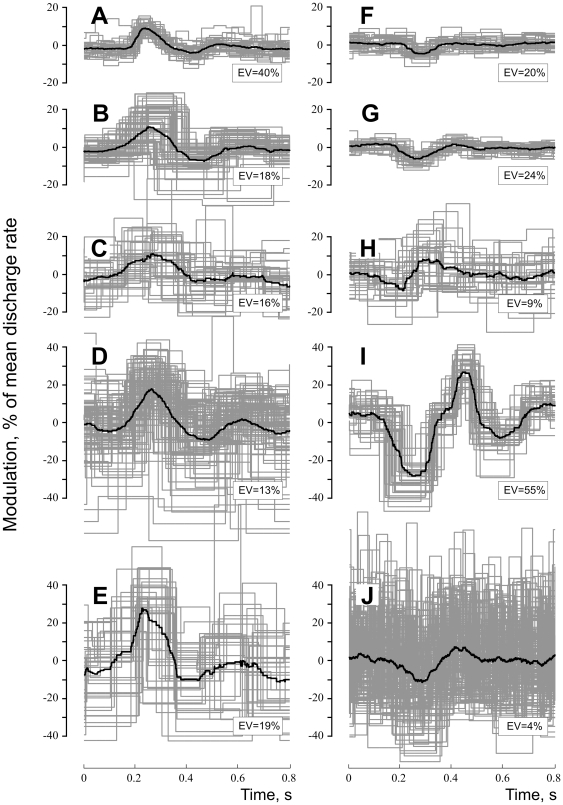
Examples of R-wave triggered discharge rate averages in pulse-wave modulated muscle spindles. Panels on the left (**A**–**E**) illustrate five afferents showing positive peak (excitatory response to the upbeat of pulse wave). Panels on the right (**F**–**J**) show five afferents displaying an initial negative peak (inhibitory effect). Discharge rate modulation is expressed as % difference from mean discharge rate. Time elapsed after R-wave is shown on the x-axis. Text box indicates the relative amount of variance explained by arterial pulsations (EV). Black thick lines represent averages. Gray thin lines show overlay of individual discharge rate traces in every cardiac cycle.

**Figure 5:**
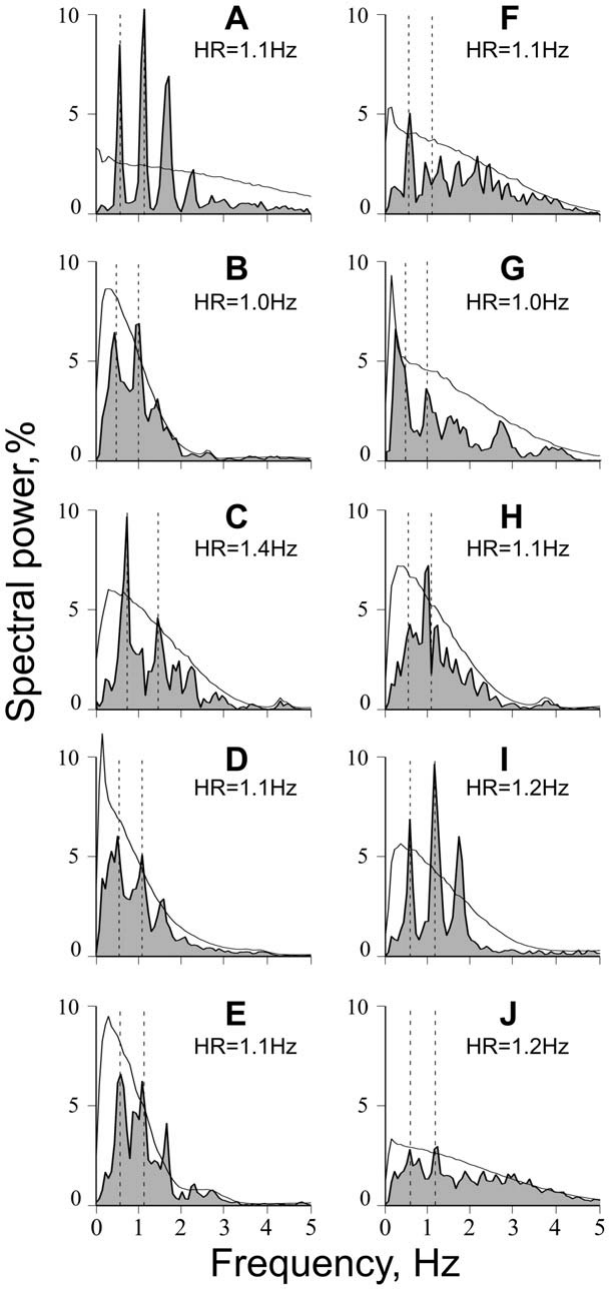
Examples of spectral analyses for the same ten afferents illustrated in **figure 4 A–J**, respectively. The gray shaded area shows the distribution of spectral power represented as a percentage of total power. Mean heart rate (HR) is indicated by the left dashed vertical line and numeric value. The right dashed vertical line indicates the frequency twice that of the heart rate. The thin black line indicates the 95% confidence interval, determined using the surrogate power spectrum distribution.

Analyses in this section refer to data obtained from 51 muscle spindle afferents discharging in the absence of active muscle contraction, i.e. in relaxed muscles. The mean discharge rate of the muscle spindles ranged from 3.5 to 23.8 imps/s (8.3 imps/s, median) and the coefficient of variation ranged from 2.0 to 84.7% (8.2%, median). For each muscle spindle afferent we calculated the R-wave triggered average to identify the modulatory effect of the pulse wave on discharge frequency. Figure 4 shows examples of individual traces and averages from 10 muscle spindle afferents (eight innervating primary and two secondary muscle spindle endings). Five of those (A–E) showed increases in discharge rate (positive modulation) at the time of the upbeat of the pulse wave, while five showed an initial decrease (F–J). Note that some afferents had a biphasic modulation profile. Figure 5 shows the equivalent analysis in the frequency domain for the same afferents. The power spectra revealed significant peaks at the cardiac frequency and its harmonics, reflecting periodic modulations of discharge rate at the heart rate frequency. The higher harmonics represent the non-sinusoidal modulation as evidenced in Figure 4. To assess the amount of pulse wave modulation we expressed the variance of the R-wave triggered average as a ratio of the total variance of the discharge rate. The amount of variance explained by arterial pulsations ranged from 0.3 to 61.7% (4.2% median). In 16 out of 51 muscle spindle afferents the variance explained by arterial pulsations was higher that 10%. To statistically assess whether the discharge rate modulation effect was indeed caused by the arterial pulsation - and not by other periodic components in the signal - the level of explained variance was compared to a set of permuted surrogate data (see Methods).

### Afferents with statistically significant pulse wave modulation

In 53% (27/51) of the afferents, pulse wave modulation was significant when evaluated against possible random effects. This effect was statistically significant in 60% (18/30) of the tested Ia afferents and in 43% (9/21) of the group II muscle spindle afferents. For the Ia afferents, the pulse modulation on average accounted for 19.1% of the variance in discharge rate; for group II afferents this was 9.4% (medians 15.3 vs. 7.6%). The largest modulatory effect within the cardiac circle peaked at about 225–300 ms (median 268 ms) after the R-wave. The mean amplitude of the peak modulation was 10.5% of the mean discharge rate. For the group Ia afferents the peak modulation was 14.3%, while for group II afferents it was 2.7% in (corresponding medians for group Ia and II afferents were 9.1% and 3.0% respectively). Regardless of the afferent type, the same number of afferents showed a positive or negative initial peak (14 vs. 13 afferents respectively). In 70% (19/27) of the afferents the modulation was biphasic and the initial peak was followed by a second peak of opposite polarity. The median amplitude of the second peak was 68% of the initial peak.

The relative size of the peak modulation correlated inversely with the mean discharge rate (r_s_ = −0.54, p<0.05), while the absolute size of modulation expressed as imp/s was not influenced by discharge rate (r_s_ = −0.13, p>0.05); this indicates that pulse wave modulation was largely additive. The relative size of the peak modulation showed a positive correlation with the coefficient of variation of discharge rate (r_s_ = 0.80, p<0.05; Spearman's rank correlation test). There was a weak relationship between cardiovascular parameters and the size of the modulatory effect across different subjects and afferents: the relative size of the peak modulation showed a weak correlation with mean blood pressure (r_s_ = 0.46, p<0.05), but not with pulse pressure (r_s_ = 0.15, p>0.05). The amount of variance explained by arterial pulsations was not correlated either with mean discharge rate (r_s_ = −0.10, p>0.05) or coefficient of variance (r_s_ = 0.33, p>0.05).

### Effects at the level of muscle spindle afferent population assessed by PCA

**Figure 6:**
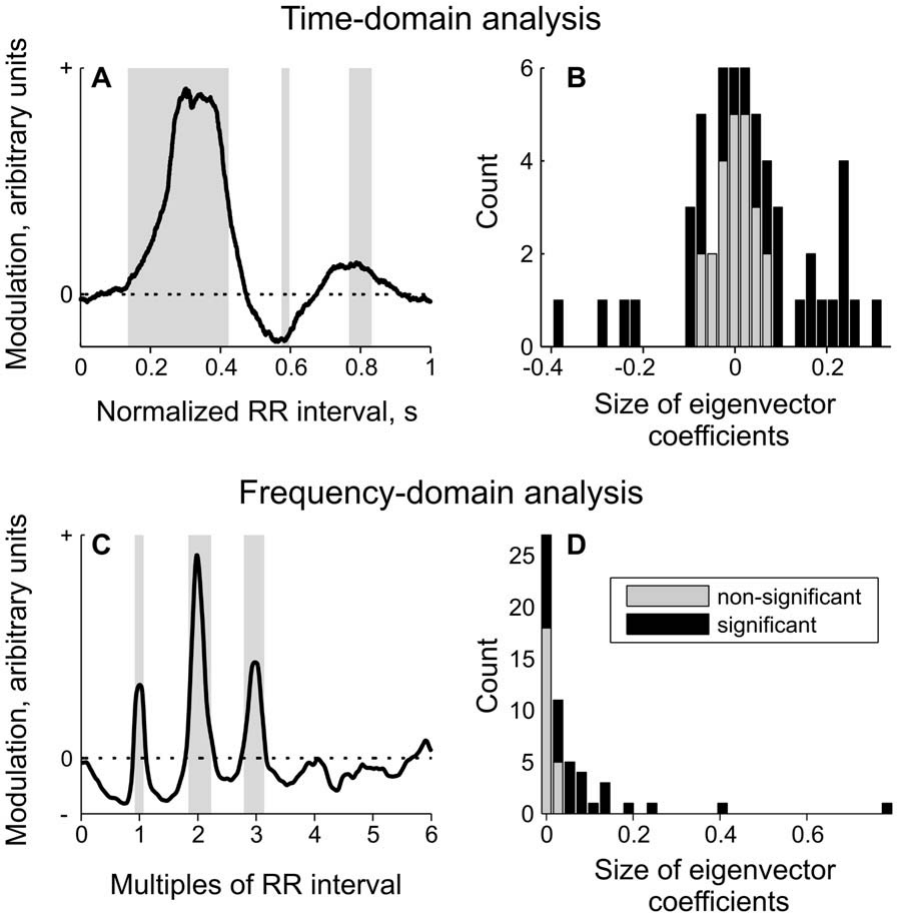
Principal component analysis (PCA) of 55 spontaneously active afferents in control (resting) conditions. Top panels display the first principal component of the time-domain analysis (explained variance = 48%), i.e. R-wave triggered discharge rate averages, extracting the modulation pattern common across afferents. Panel **A** shows the projection of the data with characteristic regions that are stable (p<0.01) across recordings depicted in gray. The time axis is normalized to the average RR-interval for each afferent before performing PCA. Panel **B** shows the histogram of the eigenvector coefficients representing the strength of this pattern in each afferent. Afferents with significant pulse wave modulation are depicted in black, non-significant afferents in grey. Lower panels display the first principal component of the frequency-domain analysis (explained variance = 43%), representing the common power spectrum across afferents (Panel **C**). The frequency axis is normalized to the average heart rate for each afferent before performing PCA. Panels **D** shows the histogram of the corresponding eigenvector coefficients.

To identify common features of the modulatory effect we used principal component analysis (PCA) to compare discharge rate modulation patterns across afferents. Most of the modulatory pattern features were explained by the first principal component (p<0.05): 48% of modulatory effect was accounted by the same pattern sharing common features between different afferents (Fig. 6A). These analyses revealed a tri-phasic modulation pattern with three stable peaks reflecting increases and decreases in discharge rate. The distribution of the corresponding eigenvector coefficients confirmed that the arterial pulse wave had either an excitatory or an inhibitory effect on different afferents, as reflected by a positive and negative coefficient, respectively (Fig. 6B). Presumably, the orientation of the muscle spindle ending with respect to a nearby blood vessel may result in loading (stretch), thereby increasing its firing rate, or unloading, and hence decreasing its discharge rate. The second principal component explained 21% of the variance (not shown) and was a modulation of the first principal component that captured the difference in latency of the modulation effect across afferents.

PCA of the power spectra further demonstrated the effect of arterial pulsations on muscle spindle discharge at the population level. The first principal component (p<0.05) explained 43% of the variance and revealed stable periodic modulation at the heart rate frequency and its harmonics (2*f* and 3*f*). The second principal component (explained variance 12%) was again a modulation of the first principal component, reflecting differences between afferents (not shown). For both time- and frequency-domain analyses, the afferents showing significant pulse wave modulation (determined from data resampling using circular permutation, as described in Methods) had larger eigenvector coefficients, as illustrated by the black histograms in Fig. 6BD.

#### Effect of physiological and cardiovascular challenges on the nature of pulse wave modulation of muscle spindles

An important question we intended to address is how much the strength of the pulse wave modulatory effect changes during physiological challenges that cause specific changes in cardiovascular parameters, sympathetic outflow or gamma motor neuron activity. In the following section our focus is on possible population effects and converging inputs from muscle spindles. Accordingly, we primarily report mean values while acknowledging that distributions might be skewed (see Methods).

### Inspiratory capacity apnoea

This physiological manoeuvre - a maximal inspiratory breath-hold - is known to cause a sustained increase in muscle sympathetic nerve activity. The manoeuvre typically caused an initial fall in systolic blood pressure typically for up to 25 mmHg and a sustained fall in pulse pressure by 9 mmHg in average.

**Figure 7:**
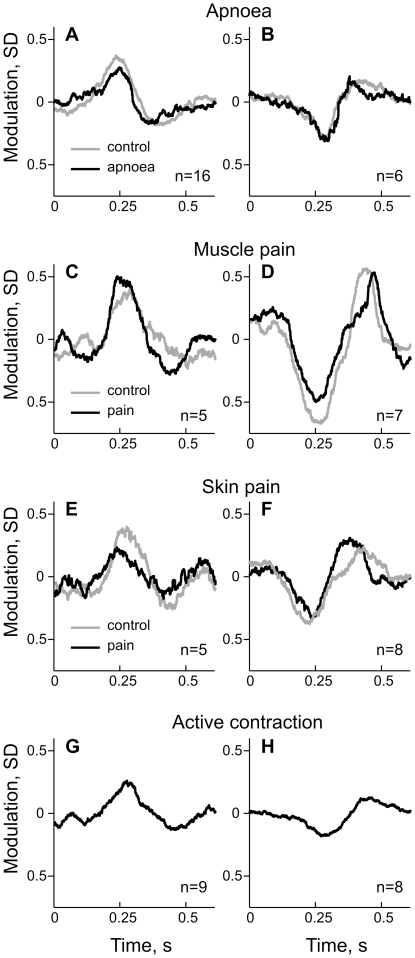
Comparison of R-wave triggered discharge rate averages between different physiological conditions. Modulation of discharge rate is normalized to the variance in discharge rate over the whole recording and plotted as a function of time, measured from the R-wave of ECG signal. Afferents are grouped according to whether the initial modulatory peak in response to the upbeat of the pulse wave is positive (graphs in left column) or negative (graphs in right column), as revealed by the sign of the eigenvector coefficient (Fig. 6**B**). **A** & **B**, changes in discharge rate modulation during inspiratory-capacity apnoea. **C** & **D**, changes in discharge rate modulation during muscle pain. **E** & **F**, changes in discharge rate modulation during skin pain. **G** & **H**, discharge rate modulation during active voluntary contraction.

Muscle spindle discharge parameters were quantified during 30 s intervals measured in 22 spindle afferents during rest and apnoea. Ongoing discharge rate and discharge variability of muscle spindles were not influenced by the manoeuvre. This was true for nine primary and thirteen secondary afferents analysed separately (p>0.05; Wilcoxon test). Moreover, the overall size of discharge rate variability explained by arterial pulsations did not change (7.0 vs. 7.4% during control condition and apnoea respectively; p>0.05 Wilcoxon test), nor did modulation strength of single afferents (p>0.05; Wilcoxon test). Indeed, the average discharge modulation was similar during the apnoea and control period for both positively (Fig. 7A) and negatively modulated afferents (Fig. 7B). There were no differences when group Ia and II afferents were analysed separately. Also, the strength of peak modulation was not influenced by the manoeuvre (6.0% vs. 6.1%; p>0.05; Wilcoxon test). The same conclusion was supported by analyses conducted on only those afferents that were significantly modulated (9/22) were selected: neither the amount of explained variance (14.1 vs. 15.1%) nor the size of peak modulation (8.6 vs. 8.3%, for control and apnoea respectively) were influenced by the apnoea (p>0.05; Wilcoxon test).

### Acute pain

We investigated whether pulse wave modulation was affected by acute muscle or skin pain. In 12 afferents investigated with muscle pain eight afferents showed significant modulation by arterial pulsations. Thirteen afferents were tested with skin pain, from which seven afferents were significantly modulated. On average, there was a small decrease in discharge rate during muscle pain (9.9 vs. 9.4 imp/s) and there was no effect of skin pain on the mean discharge rate of the muscle spindles (9.6 vs. 9.5 imp/s). The amount of variance explained by arterial pulsations was not affected by muscle pain (16.0% vs. 15.6% for control and pain respectively; p>0.05, n = 12; Wilcoxon). With skin pain the amount of variance explained by arterial pulsations on average decreased from 8.3 to 5.3%. A decrease was observed for eight out of 13 afferents but the overall effect was not significant at the population level (p>0.05; Wilcoxon; Fig. 7EF). However, from seven significantly modulated afferents the amount of explained variance decreased in six afferents and this effect was significant (p<0.05, n = 7; Wilcoxon). No changes were detected in the amount of overall discharge variability between background and muscle- or skin-pain conditions (p>0.05; Wilcoxon). Changes in the pulse wave modulatory effect observed during the pain were not correlated with changes in heart rate (p>0.05) or changes in pulse pressure (p>0.05, n = 25; Pearson correlation). In sum, these analyses indicate that no or only very weak effects from muscle or skin pain could be detected.

#### Active contraction

For seventeen muscle spindle afferents (14 primary and 3 secondary) cardiac rhythmicity was investigated during moderate strength voluntary muscle contraction. Twelve afferents (71%) showed significant modulation of discharge rate by arterial pulsations. All afferents were silent in relaxed muscles, but became engaged during active contraction most likely due to co-activation of gamma (fusimotor) neurones. Mean discharge rate during the active muscle contraction was comparable to the mean discharge rate observed in spontaneously active muscle spindles (10.2 vs. 9.6 imp/s, respectively); however, the coefficient of variance was higher during active contraction (21.7 vs. 10.8%). Due to this higher overall discharge variability the size of the explained variance was relatively small (Fig. 7GH). That is, only 2.4% of total variance was explained by arterial pulsations. In comparison, for spontaneously active afferents in relaxed muscles the arterial pulsations accounted for 9.4% (n = 51) of the total variance. During active contraction the size of the net modulatory effect at the population level was considerable - at the peak it was 8.0% of the mean discharge rate. For comparison, the strength of discharge rate modulation in afferents responding to static stretch was 7.2%.

## Discussion

We have shown that the majority of human muscle spindle afferents show a pulse-wave modulation of their discharge rate – both in relaxed muscles and during contraction. Pulse wave modulation was present in both primary and secondary muscle spindle afferents, although it was less frequent for secondary spindle endings. We describe three types of pulse wave influences on afferent discharge: (i) *muscle spindles driven by arterial pulsation*s – afferents responding exclusively to the pulse wave with every spike phase-locked to the cardiac cycle, (ii) *muscle spindles phase-locked by arterial pulsations* – afferents in which spike timing of background activity becomes phase-locked to the cardiac cycle, and (iii) *pulse wave modulated muscle spindles* - afferents in which arterial pulsations increase or decrease the discharge rate of ongoing background activity while individual spikes are not phase-locked to the arterial pulsations. It has to be emphasised, though, that in certain conditions a given muscle spindle afferent may change its behaviour. For example, muscle spindles *driven* by arterial pulsations in relaxed muscle may become *phase-locked* or *pulse-wave modulated* when background activity is evoked by muscle stretch.

Muscle spindles that were driven by arterial pulsations typically responded with one or two spikes at the time of the systolic pressure peak, while some afferents also responded to the dicrotic notch. In our sample of afferents we encountered spontaneously active Ia afferents in which the pulse wave caused ongoing spontaneous activity to phase-lock spikes to the cardiac cycle. This is the type of theoretical possibility McKeon and Burke [4] referred to as “resetting”; however, they were unable to find any muscle spindle showing this kind of behaviour. No such afferents have been previously reported in the literature, indicating that they are not common; however, they may remain largely undetected unless they are specifically searched for. The importance here is that muscle spindle afferents showing this type of behaviour potentially may pose problems for the application of spike-triggered averaging methods used to determine the synaptic connectivity between muscle spindle afferents and alpha motor neurons. That is, functionally independent afferents may show synchrony and phase locking to a common periodic source of input, such as cardiac rhythmicity. Of the spontaneously active afferents that showed significant pulse wave modulation (27 of 51 afferents), on average the amplitude of peak modulation was above 10% of discharge rate, explaining 15% of the total variance in discharge rate. Hence, even for modulated muscle spindles the pulse wave is a significant factor that determines their output behaviour.

### Modulation of discharge rate during apnoea and pain

One of the central questions in the current study was to evaluate the extent to which changes in hemodynamic parameters and sympathetic activity influence the strength of pulse wave modulation. These investigations were aimed at responding to speculations that changes in cardiovascular parameters may have an impact on the noise level in the discharge of muscle spindles and thus influence their proprioceptive function and even lead to clinically significant consequences. First, we tested effects caused by an inspiratory-capacity apnoea [19]. In contrast to our expectations, the apnoea did not significantly alter the depth of pulse wave modulation. This finding may seem surprising as the apnoea is a very potent stimulus which causes a persistent fall in pulse pressure, a sustained increase in muscle sympathetic nerve activity and a decrease in muscle blood flow. However, this finding agrees somewhat with previous observations of McKeon and Burke [4], who could not fully eliminate cardiac rhytmicity in muscle spindle discharge even when occluding arteries by cuff inflation. One of the explanations may be related to the fact that conductance of the vessel increases in proportion to the fourth power of its diameter and there might be little change in geometry between the muscle spindle organ and the neighbouring blood vessels. Also, local biomechanical and physiological mechanisms are likely to contribute to dampening the effect of changes in systemic blood pressure. On the other hand, those observations also suggest that even very strong increases in muscle sympathetic nerve activity have no direct effect on the sensitivity of muscle spindles. This confirms previous findings in humans [12], but differs from observations obtained in anaesthetised cats [13], [14]. Our findings indicate that pulse wave modulation of muscle spindle discharge is robust and relatively insensitive to spontaneous fluctuations in blood pressure. Apparently, they do not show any direct response to activation of sympathetic efferents, for which there is some evidence of direct innervation of muscle spindles [33], [34]. Any major changes in muscle spindle discharge modulation by arterial pulsations will thus most likely reflect changes in activation of gamma motor neurones. This is an important conclusion, long sought after by researchers investigating various effects of autonomic and emotional systems on proprioceptive function; pain is one such example, given its widespread effects. Furthermore, from a methodological point of view this suggests that cardiac modulation of muscle spindles could be used as a reliable indicator of muscle spindle sensitivity to dynamic stimuli. The advantage of such a weak but reliable natural dynamic stimulus is its robustness, as it does not cause any mechanical changes in muscle properties – unlike the repetitive muscle stretch typically employed to assess dynamic stretch sensitivity.

We exploited this feature to detect subtle changes in dynamic sensitivity of muscle spindles during acute muscle or skin pain. Animal studies have shown that noxious inputs onto gamma motor neurones can cause an increase in the activity of muscle spindles. It has been proposed that this may cause a fusimotor-driven increase in muscle stiffness and thus may underlie the development of chronic pain syndromes [35]. Our previous studies did not find any increase in static stretch sensitivity of muscle spindles; rather, the overall discharge rate actually decreased during muscle pain by 6%, but remained essentially the same during skin pain [16]. Irrespective of the type of pain, discharge variability parameters were not influenced. In that study, we concluded that, contrary to the “vicious cycle” hypothesis [35], acute activation of muscle or cutaneous nociceptors does not cause a reflex increase in static fusimotor drive in humans. Analyses of pulse wave modulation during acute experimental pain may provide valuable additional information: it would disclose any changes in dynamic fusimotor drive which were not specifically addressed in our previous study, partly because we wanted to avoid any subtle mechanical effects due to repetitive muscle stretching.

Changes in fusimotor activity may have significant consequences on proprioceptive acuity [36], [37], [38]. Similarly, changes in pulse wave modulation may have physiological consequences: firstly, it may be regarded as a change in noise level, and, secondly, such modulation may cause population-wide synchronised fluctuations of discharge rate, thereby affecting the excitability of, for example, the alpha motoneurone pool. Nevertheless, no changes were observed: pulse wave modulation of muscle spindle discharge did not change during nociceptive stimulation, indicating that the level of dynamic gamma-motor drive was not changed. We conclude that, while our previous study did not find any changes in muscle spindle *static* sensitivity, the current study indicates that *dynamic* sensitivity was also unchanged.

### Does pulse wave modulation influence muscle spindle function?

#### Effects on muscle spindle population input

It was important to find out whether cardiac rhythmicity is also present during active contractions. One might expect this to be the case, given that gamma-motor activity increases the stretch sensitivity of muscle spindles. However, it should also be acknowledged that the profound variability of muscle spindle discharge during voluntary muscle contraction could mask this effect. We performed analyses on muscle spindle afferents that showed no spontaneous activity, but became activated during homonymous muscle contraction due to gamma-motor co-activation. Our results showed that arterial pulsations could be easily detected during voluntary muscle contraction and the size of the modulation relative to the mean discharge rate was comparable to that found during static stretch.

Concurrent modulation of muscle spindle response by the same source in theory may be responsible for low-frequency oscillations in alpha motoneurone excitability. However, detailed analyses of the envelope of the modulatory effect revealed that there was a similar number of muscle spindles that were either excited or inhibited by the arterial pulse wave. This implies that, after signal integration at the population level, arterial pulsations of opposite polarity would largely cancel each other out. This important functional effect has been previously overlooked and therefore cardiac modulation was assumed to have a detrimental effect on the signalling capacity of muscle spindles. In contrast, our results indicate that the effect of cardiac pulsations on the population response of muscle spindles is rather limited.

#### Detection thresholds of single muscle spindle afferents

Fusimotor activation is the key factor regulating the sensitivity and dynamic range of muscle spindle operation [39], however, for detection of very weak stimuli this mechanism may not be always efficient because weak activation and twitches of intrafusal muscle fibres below their fusion frequency generate twitch induced spiking activity that may mask the effects of very weak stimuli. However, more subtle fusimotor related enhancement of muscle spindle afferent sensitivity, which is more likely to facilitate the detection of weak stimuli, are known as fusimotor after-effects [40]–[42]. The advantage of this mechanism is that it can increase sensitivity of muscle spindle endings in the absence of noise inducing fusimotor contraction.

We would like to consider another hypothetical mechanisms as to how the detection of weak stimuli can be enhanced while avoiding the adverse effect of intrafusal muscle fibre twitches. The increase of the probability of spike generation around the peak of systolic blood pressure acts as source of fluctuations, or endogenous noise source, that may facilitate the detection of stimuli that are close to threshold [11]. The underlying principle that noise could be beneficial for signal detection bears some similarity with the mechanism of stochastic resonance, which has been described for muscle spindles [43] and other types of mechanoreceptors [44], [45]. Sub-threshold signals in sensory afferents, by definition, have no effect on the output of the system, while noise can have an additive effect to the stimulus and cause subthreshold inputs to reach detection threshold. Obviously, the likelihood of this happening is higher the closer the inputs are to the threshold. Unlike high frequency stochastic noise, arterial pulsations have long well-defined period lengths that are synchronised across the whole muscle spindle population. Thus, any physiological effect mediated by arterial pulsations could be even more prominent because the probability of spike generation in response to subthreshold stimuli is increased in all excited afferents at the same time. Such a synchronised barrage of spikes generated in the population of afferents will significantly improve integration of weak inputs at the postsynaptic membrane of the 2nd order neurones. Input from muscle spindle afferents is known to converge in large numbers on the same motor neurone [46]; it has also been suggested that multiple simultaneous muscle spindle activation is required to evoke conscious sensation [47]. Thus, single spikes generated in a population of muscle spindles at random times may be too weak to be detected and to initiate any response, while spikes generated by a group of afferents at about the same time may depolarise the postsynaptic membrane and evoke a strong response also with relatively weak stimuli. To obtain physiological evidence for this, admittedly speculative, hypothesis is certainly beyond the scope of this study. We anticipate that this will give encouragement for future studies to assess the functional relevance of this hypothetical mechanism experimentally.

### Conclusions

We have shown that arterial pulsations have a significant effect on the output of more than 60% of muscle spindle afferents by periodically changing the spike timing or discharge rate. The discharge of these afferents was either completely driven by, phase-locked to, or modulated by the arterial pulsation. It is not unreasonable to expect that such considerable modulatory effects (>10% of discharge rate at the peak) may influence proprioceptive accuracy and that those effects themselves may be influenced by physiological changes in blood pressure or blood flow. However, we found no evidence that the magnitude of the physiological noise induced by arterial pulsations was affected by any of the experimentally induced conditions affecting blood pressure and muscle sympathetic nerve activity. With respect to pain, this lack of effect on magnitude of modulation also indicates that there was no activation of dynamic fusimotor drive by nociceptive reflexes. That both positive and negative modulatory effects were observed in ongoing muscle spindle responses suggests that after signal integration at the population level the opposite sign effects of modulation may at least partly cancel each other out. On the other hand, when close-to-threshold stimuli are considered we hypothesize that an additive excitatory effect of the arterial pulsations may assist the detection of close-to-threshold stimuli in single afferents. Moreover, due to the common source of such modulation, sporadic afferent firing in response to weak stimuli may synchronise in time, creating an input that may become detectable within sensory (and sensorimotor) pathways.
